# Risk factors for PrEP and ART medication adherence challenges in cis-gender South African men who have sex with men in Johannesburg and Pretoria

**DOI:** 10.1093/inthealth/ihae090

**Published:** 2025-02-13

**Authors:** Jacqueline Pienaar, Lindiwe Tsope, Mapaseka Mabena, Pontsho Komane, Maria Sibanyoni, Boitumelo Ramashala, Elizabeth Wahome, Charlene Denousse, Ankiza Gakunu, Elise M van der Elst, Danielle Giovenco, Don Operario, Eduard J Sanders

**Affiliations:** Health Systems Division, Aurum Institute, Johannesburg 2194, South Africa; Implementation Science Division, Aurum Institute, Johannesburg 2194, South Africa; Health Systems Division, Aurum Institute, Johannesburg 2194, South Africa; Health Systems Division, Aurum Institute, Johannesburg 2194, South Africa; Health Systems Division, Aurum Institute, Johannesburg 2194, South Africa; Health Systems Division, Aurum Institute, Johannesburg 2194, South Africa; Radcliffe Department of Medicine, University of Oxford, Oxford OX1 2JD, UK; Inuka Foundation, Nairobi, Kenya; Inuka Foundation, Nairobi, Kenya; Department of Interdisciplinary Social Science, Utrecht University, Utrecht, The Netherlands; Behavior, Social and Health Education, Emory University, Atlanta, GA 30322, USA; Behavior, Social and Health Education, Emory University, Atlanta, GA 30322, USA; Implementation Science Division, Aurum Institute, Johannesburg 2194, South Africa; Dunn School of Pathology, University of Oxford, Oxford OX1 3RE, UK

## Abstract

**Background:**

Mental health challenges are common among men who have sex with men (MSM) in South Africa and may impact medication adherence.

**Methods:**

We determined the prevalence and risk factors of medication adherence challenges among 160 pre-exposure prophylaxis (PrEP)- and 40 antiretroviral therapy (ART)-taking MSM registered at two key population clinics in Johannesburg and Pretoria in 2023. We used modified Poisson regression to estimate associations between participant characteristics and medication adherence challenges (missed dosage on ≥1 d in the last month).

**Results:**

A total of 106 (53.5%) participants (57.6% on PrEP, 37.5% on ART; p=0.02) had medication adherence challenges and 61 (30.5%) participants (31.2% on PrEP, 27.5% on ART; p=0.23) met criteria for moderate to severe symptoms of depression (score ≥10 on the 9-item Patient Health Questionnaire). In multivariable analysis, predictors included PrEP use (adjusted prevalence ratio [aPR]=1.81 [95% confidence interval {CI} 1.21 to 2.73), clinic in Pretoria (aPR 1.43 [95% CI 1.08 to 1.89]), transactional sex (aPR 1.81 [95% CI 1.34 to 2.44]), moderate to severe depression (aPR 1.50 [95% CI 1.19 to 1.89]) and use of social media (aPR 1.45 [95% CI 1.05 to 2.00]).

**Conclusions:**

Depression is common and may be an important risk factor for poor medication adherence among MSM in South Africa. Future research should leverage a longitudinal study design to inform potential interventions.

## Introduction

Men who have sex with men (MSM) are one of the fastest growing human immunodeficiency virus (HIV) risk populations in South Africa, the country with the world's largest HIV epidemic.^1,2^ Common mental health problems—including depression and anxiety—are prevalent among MSM in South Africa due to social factors including exposure to anti-MSM stigma, internalized stigma, concealment of sexual identity, isolation and loneliness.^3^ There is a body of research indicating a high burden of unaddressed mental health problems among MSM globally, which can contribute to challenges across the HIV prevention and care continua, including poor adherence to daily oral pre-exposure prophylaxis (PrEP) and antiretroviral therapy (ART).^3,4^ However, most of this research is based on studies of MSM in the USA, with few studies conducted with MSM in South Africa or other parts of sub-Saharan Africa.^2^ Consequently, few specific programmatic efforts have been devoted to improving mental health as part of initiatives aimed at ending HIV among MSM in Africa.^4^

While there have been recent attempts to improve provision of HIV prevention and care programs for MSM in South Africa, including programs to promote daily PrEP or ART initiation following HIV testing,^5–7^ little is known about common mental health challenges among PrEP- or ART-taking MSM. There is compelling evidence for the need to assess the relationship between mental health and PrEP or ART adherence among MSM in this setting. Results of a meta-analysis based on 95 studies and >35 000 patients—the majority of studies conducted in the USA and none in South Africa—reported that depression is consistently associated with non-adherence to HIV treatment.^8^ Qualitative research with MSM in South Africa has described determinants of poor ART adherence, including medical mistrust about ART effects, feelings of social isolation, anticipated stigma and gossip in the general community and concerns about disclosure.^9,10^ Research has also indicated the need for targeted PrEP adherence interventions to optimize protective levels among MSM in South Africa.^6,11^ Studies in Kenya and South Africa have shown that among daily PrEP-taking MSM, less than one in five had sufficient tenofovir-diphosphate levels to confer protection (i.e. four or more daily PrEP doses taken per week).^5,12,13^

To address a growing research priority, we sought to improve empirical understanding of mental health and HIV medication adherence challenges among MSM in South Africa. In particular, we were interested to explore adherence challenges among MSM using daily PrEP and MSM using ART and then to examine depression and other mental health symptomatology as risk factors for adherence challenges. Findings from this exploratory study may help us establish a basis for MSM-focused interventions targeting the co-occurrence of mental health and HIV medication adherence.^14^

## Methods

This cross-sectional study was conducted between July and October 2023. Participants were recruited from the Aurum Institute, Johannesburg, South Africa. Aurum offers HIV-related prevention and treatment support services to >20 000 members of key populations, including MSM and transgender women (TGW), at five ‘POP INN’ clinics in South Africa. Clients enrolled at Aurum receive free PrEP or ART, adherence counselling, tests for HIV every 3 months for those on PrEP, screening for sexually transmitted infections and tuberculosis and psychosocial support services. Clients have the option to participate in monthly peer-led support group meetings, typically comprising 8–10 MSM and/or trans-community members, where they can talk about health-related experiences and challenges. The clinics also provide free Wi-Fi, access to computers and a comfortable space to relax and connect with peers.

A total of 160 daily PrEP- and 40 ART-taking participants were recruited while attending follow-up PrEP or ART refill appointments at Aurum's Tshwane (Pretoria) and Ekurhuleni (Johannesburg) POP INN clinics. Eligible participants were English speaking, self-identified MSM (defined as men who reported engaging in sex with a man within the past year) and receiving daily PrEP or ART services at a POP INN clinic in Tshwane (Pretoria) or Ekurhuleni (Johannesburg). Participants were ineligible if they were presently engaged in mental health therapy. Once determined eligible, they were invited to complete a questionnaire that assessed sociodemographic characteristics, mental health and behaviour factors administered via computer-assisted self-interview software. Participants were compensated 200 Rand (approximately US$10). Participants were asked to debrief with a counsellor after completing the survey to identify any problems or questions that had been upsetting.

### Measures

Participants were asked about age, education, race, employment, transactional sex and daily PrEP and ART adherence using questions from the HIV Prevention Trials Network 075 study among MSM in South Africa.^15^ We defined medication adherence challenges as missing taking PrEP or ART on ≥1 d in the past 30 d.

#### Depression

The 9-item Patient Health Questionnaire (PHQ-9) measures the frequency of depression symptoms within the past 2 weeks. The PHQ-9 comprises five categories, where a 0–4 indicates no depressive symptoms, 5–9 mild depressive symptoms, 10–14 moderate depressive symptoms, 15–19 moderately severe depressive symptoms and 20–27 severe depressive symptoms.^16^ This scale has been used previously in MSM populations in sub-Saharan Africa (SSA), including MSM living with HIV.^17,18^ We assessed moderate to severe (vs minimal to mild) depression using a cut-off ≥10.

#### Trauma

Recent trauma (within the last year) was assessed using the four-item USAID Health Policy Initiative MSM Trauma Screening Tool, which inquired about sexual, physical, verbal and psychological trauma.^19^ We assessed each type of trauma separately.

#### Anxiety

The 7-item Generalized Anxiety Disorder (GAD-7) scale measures symptoms of anxiety with a range of 0–21.^20^ The GAD-7 score comprises four categories, where a cut-off of 0–4 indicates minimal anxiety, 5–9 mild anxiety, 10–14 moderate anxiety and 15–21 severe anxiety. The scale has been extensively used, including in SSA.^20,21^ We assessed moderate to severe (vs minimal to mild anxiety) using a cut-off ≥10.

#### Alcohol use

Alcohol abuse was measured using the Alcohol Use Disorder Identification Test (AUDIT), a validated instrument developed for international settings.^22^ The survey has 10 questions, scored 0–4, with potential scores ranging from 0 to 40. A score of at least 8 was considered indicative of hazardous drinking.^22^

#### Substance use

The 10-item Drug Abuse Screening Test (DAST-10) was used to measure problematic use of non-prescription substances other than alcohol or tobacco. The survey consists of 10 ‘yes/no’ questions with scores ranging from 0 to 10. A validated cut-off score ≥3 was used to identify harmful substance use.^5^

#### Childhood abuse

Childhood abuse was measured using the four-item Childhood Experience of Care and Abuse scale.^23^ A positive response to any item was classified as childhood abuse.

#### Sexual stigma

Sexual stigma was measured using an abridged version of the modified China MSM Stigma Scale by Logie et al.^24^ The scale measures two dimensions: perceived stigma, or the perception of social censure of homosexuality, and enacted stigma, the direct experience of homophobia. It consists of 11 questions scored 0–3 (never, once or twice, a few times, many times). Potential scores range from 0 to 33, with a higher score representing greater perceived sexual stigma.

#### Social network and social media use

Social network was assessed through social media use (e.g. WhatsApp or Facebook) in the past month, including specific gay applications (Grindr) and if a mobile phone was used to access social media.

### Statistical analysis

For categorical variables, frequency and percentage were calculated and Pearson's χ^2^ test was performed for comparisons across PrEP- or ART-taking participants (i.e. HIV status). For continuous variables, median and interquartile range (IQR) were used to compare across HIV status. We fit modified Poisson regression with robust error variances to estimate prevalence ratios (PRs) and their 95% confidence intervals (CIs) for adherence challenges. Factors associated with adherence challenges in bivariable analysis at an α level of 0.1 were included in the initial multivariable model. The final multivariable model retained only predictors associated with adherence challenges at an α of 0.1 in the initial multivariable model adjusted for age. Statistical significance was set at p≤0.05. Data were analysed using Stata 18.0 (StataCorp, College Station, TX, USA).

## Results

A total of 200 enrolled participants (160 on PrEP and 40 on ART) completed the survey (Table 1). The median age was 31.4 y, most participants were black (90.0%), South African (88.5%), had completed secondary education (50.5%) or tertiary or higher education (46.5%) and approximately 70% had some form of employment. Participants in Pretoria vs Johannesburg were younger, had higher tertiary education, were more often a student, included more white people and a larger proportion used social media (details in Supplementary Table S1).

**Table 1.** Distribution of characteristics of daily PrEP- and ART-taking cis-gender MSM, Aurum POP INN clinics in Johannesburg and Pretoria, 2023

Characteristics	Overall (N=200)	PrEP (n=160)	ART (n=40)^a^	*P*-value^b^
Age (years), median (IQR)	31.4 (26.4–37.4)	31.0 (26.2–36.5)	34.0 (27.1–39.6)	0.28
Ever married to a female, n (%)	29 (14.5)	26 (16.3)	3 (7.5)	0.16
Education, n (%)				0.97
Primary	6 (3.0)	5 (2.5)	1 (3.1)	
Secondary	101 (50.5)	81 (50.0)	20 (50.6)	
Higher/tertiary/other	93 (46.5)	74 (46.3)	19 (47.5)	
Race, n (%)				0.38
Black	180 (90.0)	143 (89.4)	37 (92.5)	
White	11 (5.5)	10 (6.3)	1 (2.5)	
Coloured	5 (2.5)	3 (1.9)	2 (5.0)	
Indian/Asian	4 (2.0)	4 (2.5)	0 (0.0)	
Nationality, n (%)				0.59
South African	177 (88.5)	143 (89.4)	34 (85.0)	
Zimbabwean	17 (8.5)	12 (7.5)	5 (12.5)	
Other	6 (3.0)	5 (3.1)	1 (2.5)	
Employment, n (%)				0.39
Unemployed	61 (30.5)	51 (31.9)	10 (25.0)	
Employed (full time)	84 (42.0)	63 (52.0)	21 (39.4)	
Employed (part time/casual)	29 (14.5)	23 (15.0)	6 (14.4)	
Self-employed	26 (13.0)	23 (14.4)	3 (7.5)	
Received payment for sex, past year^c^, n (%)	16 (8.0)	8 (5.0)	8 (20.0)	**0.001**
Paid for sex, past year^b^, n (%)	17 (22.5)	12 (16.9)	5 (45.0)	0.31
Group sex, past year, n (%)	38 (19.0)	27 (16.9)	11 (16.9)	0.12
Medication adherence, n (%)				
Miss taking daily PrEP/ART on ≥1 d	106 (53.5)	91 (57.6)	15 (37.5)	**0.02**
Trauma, n (%)				
Forced or coerced sex	15 (7.5)	11 (6.9)	4 (10.0)	0.50
Physical abuse	45 (53.5)	27 (57.6)	18 (37.5)	**<0.001**
Verbal abuse	93 (46.5)	72 (45.0)	21 (52.5)	0.40
Threats or intimidation	65 (32.5)	50 (31.3)	15 (37.5)	0.45
Any childhood abuse	103 (51.5)	82 (51.3)	21 (52.5)	0.89
Depressive symptoms (PHQ-9), past 2 weeks, n (%)				0.23
Minimal to mild (0–9)	139 (69.5)	110 (68.8)	29 (72.5)	
Moderate to severe (10–27)	61 (30.5)	50 (31.2)	11 (27.5)	
Anxiety (GAD-7), past 2 weeks, n (%)				0.28
Minimal to mild (0–9)	153 (67.5)	125 (76.1)	28 (70.0)	
Moderate to severe (10–21)	47 (30.5)	35 (23.9)	12 (30.0)	
Disordered alcohol use (AUDIT), past year, n (%)				0.47
Low (0–7)	125 (62.5)	102 (63.8)	23 (57.5)	
Hazardous (8–40)	75 (37.5)	58 (36.3)	17 (42.5)	
Problematic substance use (DAST-10), past year, n (%)				0.19
No (0–2)	181 (90.5)	147 (91.9)	34 (85.0)	
Yes (≥3)	19 (9.5)	13 (8.1)	6 (15.0)	
Sexual stigma score (0–33), median (IQR)	9 (5–13)	8 (5–14)	9 (6–13)	0.97
Incarcerated in the past 3 months, n (%)				
No	194 (97.0)	138 (99.3)	56 (91.8)	**0.004**
Yes	6 (3.0)	1 (0.7)	5 (8.2)	
Used social network applications to communicate with peers, n (%)				
No	71 (35.5)	56 (35.0)	15 (37.5)	0.5
Yes	129 (64.5)	104 (65.0)	25 (62.5)	
Used mobile phone to access social media services or gay apps^c^, n (%)				
No	70 (35.5)	58 (36.9)	12 (30.0)	0.4
Yes	127 (64.5)	99 (63.1)	28 (70.0)	

a For 39 ART-taking participants whose recent viral load was available, 5 of 20 (25%) in Tshwane and 3 of 19 (16%) in Ekurhuleni (p=0.48) had a viral load ≥200 copies/ml.

b Two-sided value based on χ^2^ test for proportions or Wilcoxon rank sum test.

c Missing three values for transactional sex and mobile phone categories.

Bold values indicate variables whose p-values are ≤0.05.

Overall, 22.5% paid for sex during the past year and 8% reported receiving payment for sex in the past year, with more ART patients than PrEP patients reporting receiving payment for sex (5.0% on PrEP vs 20.0% on ART; p=0.001).

A total of 106 (53.5%) participants (57.6% on PrEP, 37.5% on ART; p=0.02) reporting medication adherence challenges (i.e. missed PrEP or ART on ≥1 d in the last month). For 39 ART-taking participants whose recent viral load was available, 5 of 20 (25%) in Tshwane and 3 of 19 (16%) in Ekurhuleni (p=0.48) had a viral load ≥200 copies/ml. Physical abuse was reported more frequently by participants on PrEP than on ART (57.6% vs 37.5%; p=0.001). Hazardous alcohol use (AUDIT score ≥8; 37.5% of respondents), psychological trauma and any childhood abuse (32.5% and 51.5%, respectively, of respondents) and symptoms of moderate to severe anxiety (GAD-7 ≥10; 30.5% of respondents) were not significantly different between PrEP- and ART-taking participants. Few participants (3%) had been incarcerated in the last 3 months, though this differed between groups (0.7% on PrEP vs 8.2% on ART; p=0004).

Sixty-one (30.5%) participants (31.2% on PrEP, 27.5% on ART; p=0.23) met criteria for moderate to severe symptoms of depression (PHQ-9 score ≥10).

In bivariable analysis, medication adherence challenges were associated with PrEP vs ART use (p=0.047), clinic in Pretoria (p<0.001), receiving payment for sex (p=0.003), experiencing verbal abuse (p=0.038), moderate to severe symptoms of depression (p<0.001), hazardous alcohol use (p=0.011), problematic substance use (p=0.021), greater sexual stigma score (p<0.001), using social network to communicate with peers (p=0.050) and using a mobile phone to access social media services or gay apps (p=0.002) (Table 2).

**Table 2.** Risk factors associated with medication adherence challenges in cis-gender daily PrEP- and ART-taking MSM, Aurum POP INN clinics in Johannesburg and Pretoria, 2023.

	Bivariable analysis		Multivariable analysis	
Characteristics	PR (95% CI)	p-Value	aPR (95% CI)	p-Value
Medication				
ART	Reference		Reference	
PrEP	**1.54 (1.01 to 2.34)**	**0.047**	**1.81 (1.21 to 2.73)**	**0.004**
Age	0.99 (0.98 to 1.01)	0.490	1.01 (0.99 to 1.02)	0.246
Ever married to a female	0.80 (0.55 to 1.18)	0.262		
Education				
Primary	Reference			
Secondary	0.92 (0.40 to 2.11)	0.844		
Tertiary	1.24 (0.55 to 2.81)	0.607		
Race				
Black	Reference			
White	1.04 (0.60 to 1.83)	0.880	NI^a^	
Coloured	1.53 (0.97 to 2.43)	0.070	NI^a^	
Indian/Asian	1.44 (0.80 to 2.58)	0.225	NI^a^	
Nationality				
South African	Reference			
Zimbabwean	0.74 (0.41 to 1.33)	0.319		
Other	0.60 (0.19 to 1.88)	0.383		
District				
Ekurhuleni (Johannesburg)	Reference			
Tshwane (Pretoria)	**1.65 (1.25 to 2.18)**	**<0.001**	**1.43 (1.08 to 1.89)**	**0.012**
Employment				
Unemployed	Reference		Reference	
Employed (full time)	0.89 (0.67 to 1.19)	0.438	0.96 (0.73 to 1.25)	0.741
Employed (part time/casual)	0.65 (0.39 to 1.07)	0.091	0.64 (0.40 to 1.04)	0.072
Self-employed	0.82 (0.53 to 1.27)	0.384	0.81 (0.53 to 1.23)	0.322
Received payment for sex, past year	**1.55 (1.16 to 2.07)**	**0.003**	**1.81 (1.34 to 2.44)**	**<0.001**
Paid for sex, past year	0.64 (0.33 to 1.23)	0.182		
Group sex, past year	1.08 (0.78 to 1.48)	0.655		
Trauma				
Sexual	0.79 (0.42 to 1.47)	0.453		
Physical	0.92 (0.66 to 1.27)	0.605		
Verbal	**1.31 (1.02 to 1.71)**	**0.038**	NI^a^	
Psychological	1.15 (0.88 to 1.50)	0.305		
Any childhood abuse	1.21 (0.93 to 1.57)	0.165		
Depressive symptoms (PHQ-9), past 2 weeks				
Minimal to mild (0–9)	Reference		Reference	
Moderate to severe (10–27)	**1.60 (1.26 to 2.05)**	**<0.001**	**1.50 (1.19 to 1.89)**	**0.001**
Anxiety (GAD-7), past 2 weeks				
Minimal to mild (0–9)	Reference			
Moderate to severe (10–21)	1.21 (0.92 to 1.59)	0.174		
Disordered alcohol use (AUDIT), past year				
Low (0–7)	Reference			
Hazardous (8–40)	**1.39 (1.08 to 1.78)**	**0.011**	NI^a^	
Problematic substance use (DAST-10), past year				
No (0–2)	Reference			
Yes (≥3)	**1.43 (1.06 to 1.94)**	**0.021**	NI^a^	
Sexual stigma score (0–33)	**1.04 (1.02 to 1.06)**	**<0.001**	NI^a^	
Incarcerated in the past 3 months				
No	Reference			
Yes	1.25 (0.70 to 2.25)	0.445		
Used social network to communicate with peers				
No	Reference			
Yes	**1.35 (1.00 to 1.83)**	**0.050**	NI^a^	
Used mobile phone to access social media services or gay apps				
No	**Reference**			
Yes	**1.73 (1.23 to 2.44)**	**0.002**	**1.45 (1.05 to 2.00)**	**0.023**

Bold values indicate variables whose 95% CI of the PR did not cross 1.

^a^NI: not included in the final model. Only factors significant at a category-specific p-value <0.1 in the initial multivariable model (data not shown) were retained in the final multivariable model.

In multivariable analysis, predictors of medication adherence challenges included PrEP vs ART use (adjusted prevalence ratio [aPR]=1.81 [95% CI 1.21 to 2.73]), clinic in Pretoria (aPR 1.43 [95% CI 1.08 to 1.89]), transactional sex (aPR 1.81 [95% CI 1.34 to 2.44]), moderate to severe depression (aPR 1.50 [95% CI 1.19 to 1.89]) and social media use (aPR 1.45 [95% CI 1.05 to 2.00]) in a model controlled for age.

## Discussion

Findings from this study provide additional evidence of the co-occurring challenges in HIV medical adherence and mental health among MSM seeking HIV services in South Africa. We found a high prevalence of medication adherence challenges among both PrEP- and ART-taking MSM, with notable differences between MSM clinics in Johannesburg and Pretoria as well as higher self-reported adherence challenges among MSM using PrEP. We also observed high levels of psychological challenges such as depression and anxiety, hazardous alcohol use, experiences of childhood abuse and trauma related to lifetime physical abuse, verbal abuse, threats and intimidation. In a multivariable model, depression, PrEP use, residence in Pretoria, sex work and use of a mobile phone to access social media were identified as predictors of adherence challenges. These findings suggest a complex relationship between mental health, social determinants and HIV outcomes among MSM populations^25^ that requires further research.

Our findings suggest that depression may be an important predictor of adherence challenges among South African MSM, and future research should consider the utility of tailored interventions addressing the unique needs and challenges of PrEP- and ART-taking MSM within HIV prevention and care programs. Addressing these factors through integrated mental health interventions within HIV prevention and care programs may also optimize health outcomes and reduce health disparities among MSM in South Africa. Although many HIV prevention and care programs may acknowledge person-level factors that affect medication adherence and clinical outcomes, they often do not provide clinical and evidence-based services to address patients’ mental health and psychological counselling needs—especially in high-burden and underresourced settings.^26–28^ In South Africa, as in many low- and middle-income countries, there is a limited workforce to provide these integrated services.^29,30^ Differentiated mental health and psychological services for MSM may be warranted, particularly in light of the unique social determinants of risk in this population.^3^

There is an emerging literature in SSA supporting the beneficial effects of mental health interventions provided by lay health workers (LHWs) for treating common psychosocial problems and HIV medication adherence.^14,31^ The Friendship Bench (FB), for example, is an evidence-based community mental health intervention developed and validated in multiple low-resource settings in SSA.^32–34^ A key feature of FB is the use of LHWs who are trained to deliver evidence-based problem-solving therapy for individuals screening positive for depression and anxiety.^35^ However, these programs were delivered to general community members, necessitating further program adaptation and evaluation to examine the relevance and impact of FB on MSM.

An ongoing project by our group, entitled WeCare (R34MH135806), has engaged community MSM to adapt an LHW-delivered counselling approach, based on the evidence-based FB and Inuka intervention protocols,^36^ to address the dual and interconnected priorities of mental health and medication adherence.^37^ In addition to adapting counselling content and style to address MSM's needs, this work trains lay counsellors/coaches from the MSM community in Johannesburg and Pretoria to deliver the intervention. Qualitative evaluation of trained MSM coaches indicated improvements in their counselling skills, but persistent challenges in program delivery, including the need for specialized knowledge and the high MSM community demand for services.^37^

Adaptation of the FB model to address the specific needs of MSM populations could offer a culturally sensitive and contextually appropriate approach to improving ART and PrEP adherence and mental health outcomes in South Africa. Given the status-neutral approach of the FB intervention, which focuses on addressing common mental health challenges rather than specific health conditions, it holds promise for addressing the mental health needs of both HIV-negative individuals on PrEP and HIV-positive individuals on ART within MSM communities.

Limitations of our study include a relatively small sample of English-speaking participants recruited from an established HIV service organization that provided programs to MSM and offered free daily PrEP and ART; although this is an aspirational context for many LMIC settings, the findings reported here may not generalize to all South African MSM. Our study also had a cross-sectional design and the assessment of our outcome—adherence challenges—overlapped with the assessment of several predictors, limiting the predictive ability of our model. Associations in bivariable and multivariable models should not be interpreted causally. Last, this study relied on self-reported measures of theorized predictors and our medication adherence outcome, which may be subject to recall or social desirability bias.

## Conclusions

Our study highlights risk factors for poor medication adherence among daily PrEP- and ART-taking MSM in South Africa, which include moderate to severe depression. Although identifying MSM with depression may have prognostic value, future research should leverage a longitudinal study design to establish a causal relationship between depression and adherence to inform potential interventions. Targeted interventions addressing medication adherence and depression may improve prevention and treatment outcomes and reduce health disparities among MSM populations.

## Supplementary Material

ihae090_Supplemental_File

## Data Availability

The data that support the findings of this study are available from the corresponding author upon reasonable request and approval of principal investigators.
