# Supplementary material for: Risk factors for PrEP and ART medication adherence challenges in cis-gender South African men who have sex with men in Johannesburg and Pretoria
Source: Int Health. 2025 Feb 13;17(4):509–16. doi: 10.1093/inthealth/ihae090 (PMC12212232; doi:10.1093/inthealth/ihae090)
Supplement: ihae090_Supplemental_File [file ihae090_supplemental_file.docx]

**Supplemental table 1. Distribution of socio-demographic characteristics of PrEP and ART taking cisgender MSM, by POP INN clinic in Pretoria or Johannesburg, 2023**

| **Characteristics** | | | **Overall (n = 200)** | | **Pretoria**  **(n = 100)** | | **Johannesburg**  **(n = 100)** | | ***P* value** |
| --- | --- | --- | --- | --- | --- | --- | --- | --- | --- |
|  |  |  | **n (%), or median (IQR)** | | **n (%), or median (IQR)** | | **n (%), or median (IQR)** | |  |
| Age (years) | | | 31.4 (26.4-37.4) | | 29.6 (25.1-36.1) | | 32.4 (22.7-46.5) | | 0.09 |
| Ever married to a female | | | 29 | (14.5) | 5 | (5.0) | 24 | (24.0) | **<0.001** |
| Education | | |  |  |  |  |  |  | **<0.001** |
|  | Primary | | 6 | (3.0) | 1 | (1.0) | 5 | (5.0) |  |
|  | Secondary | | 101 | (50.5) | 31 | (31.0) | 70 | (70.0) |  |
|  | Higher/tertiary/other | | 93 | (46.5) | 68 | (68.0) | 25 | (25.0) |  |
| Student | | |  |  |  |  |  |  | **<0.001** |
|  | | No | 133 | (66.5) | 41 | (41.0) | 82 | (82.0) |  |
|  | | Yes | 67 | (33.5) | 59 | (59.0) | 18 | (18.0) |  |
| Race | | |  |  |  |  |  |  | **0.004** |
|  | Black  White  Coloured  Indian/Asian | | 180  11  5  4 | (90.0)  (5.5)  (2.5)  (2.0) | 83  11  3  3 | (83.0)  (11.0)  (3.0)  (3.0) | 97  0  2  1 | (97.0)  (0.0)  (2.0)  (1.0) |  |
| Nationality | | |  |  |  |  |  |  | 0.206 |
| South African  Zimbabwe  Other | | | 177  17  6 | (88.5)  (8.5)  (3.0) | 85  12  3 | (85.0)  (12.0)  (3.0) | 92  5  3 | (92.0)  (5.0)  (3.0) |  |
| Employment | | |  |  |  |  |  |  | 0.886 |
|  | Unemployed | | 61 | (30.5) | 33 | (33.0) | 28 | (28.0) |  |
|  | Employed (full time) | | 84 | (42.0) | 41 | (41.0) | 43 | (43.0) |  |
|  | Employed (part-time / casual) | | 29 | (14.5) | 14 | (14.0) | 15 | (15.0) |  |
|  | Self-employed | | 26 | (13.0) | 12 | (12.0) | 14 | (14.0) |  |
| Used social network applications to communicate with peers | | |  |  |  |  |  |  | **<0.001** |
|  | No | | 71 | (35.5) | 19 | (19.0) | 52 | (52.0) |  |
|  | Yes | | 129 | (64.5) | 81 | (81.0) | 48 | (48.0) |  |
| Used mobile phone to access social media services or gay apps^1^ | | |  |  |  |  |  |  | **<0.001** |
| No | | | 70 | (35.5) | 18 | (18.2) | 52 | (53.1) |  |
| Yes | | | 127 | (64.5) | 81 | (81.8) | 46 | (46.9) |  |

Bold values indicate variables whose *P*-values are ≤0.05.

1. 3 values missing for mobile phone use.
